# From Specimen to Signature: A Retrospective Audit of Turnaround Times in Routine Pathology Reporting at the Department of Pathology, Bangladesh Medical University

**DOI:** 10.7759/cureus.105292

**Published:** 2026-03-16

**Authors:** Mohammad Mosiur Rahman, Ferdousy Begum, Md. Zillur Rahman, Tasmia Islam, Sanjida Parveen, Papiya Rahman, Raisa Badhan, Shahedul Islam

**Affiliations:** 1 Pathology, Bangladesh Medical University, Dhaka, BGD; 2 Obstetrics and Gynaecology, Directorate General of Health Services, Dhaka, BGD; 3 Microbiology, National Institute of Burn and Plastic Surgery, Dhaka, BGD

**Keywords:** cytopathology, direct immunofluorescence, fluorescence in situ hybridization, histopathology, immunohistochemistry, laboratory information system, turnaround time

## Abstract

Background

Turnaround time (TAT) is a critical quality indicator in diagnostic pathology, influencing clinical decision-making, patient management, and healthcare efficiency. Systematic benchmarking of TAT across routine and specialized pathology services is essential for identifying workflow strengths and performance gaps, particularly in high-volume tertiary care settings. This study aims to evaluate and benchmark TAT performance across histopathology, cytopathology, and specialized laboratory tests at the Department of Pathology, Bangladesh Medical University, and to compare observed performance against established TAT standards and compliance benchmarks.

Methodology

A retrospective audit was conducted, analyzing TAT data for histopathology, cytopathology, immunohistochemistry, immunofluorescence, and cytogenetic tests over the study period. TAT was calculated in working days from specimen receipt to final report authorization. Performance metrics included average, median, interquartile range, maximum TAT, and percentage of reports delivered within predefined target TAT. Observed performance was benchmarked against institutional and international quality standards, with compliance defined as ≥90% on-time reporting.

Results

Histopathology specimens demonstrated average TATs of seven to eight working days across large, medium, and small samples; however, compliance varied significantly by specimen size, ranging from 77.10% for large specimens to 89.51% for small specimens. Cytopathology services showed superior performance, with average TATs of three to five working days and high compliance rates (86.48%-96.22%), particularly for fluid cytology and liquid-based cytology. Specialized tests exhibited wide variability in TAT and compliance. Fluorescent in situ hybridization (FISH) achieved 100% compliance. Direct immunofluorescence (DIF) showed the lowest performance, with only 67.7% of reports delivered within the target timeframe.

Conclusions

This study highlights substantial variability in TAT performance across pathology subspecialties, driven largely by specimen complexity and test-specific workflow demands. While cytopathology and FISH diagnostic tests demonstrated excellent efficiency and compliance, large histopathology specimens and DIF represent key areas for targeted quality improvement. Continuous TAT monitoring using compliance-based and variability-sensitive metrics can support process optimization and enhance diagnostic service delivery in tertiary care pathology laboratories.

## Introduction

Timely and accurate pathology reporting is a cornerstone of modern healthcare, forming the basis for diagnosis, prognosis, and treatment decisions. The interval from receipt of a specimen in the laboratory to the final signed pathology report, commonly referred to as the turnaround time (TAT), is a critical indicator of laboratory efficiency and quality service delivery [1,2]. Delays in TAT can have direct clinical consequences, including postponed diagnoses and operative procedures, delayed treatment initiation, prolonged hospital stays, and increased patient anxiety [3]. In resource-limited settings, these consequences may be amplified due to constraints in infrastructure, staffing, and workflow organization.

Benchmarking TAT has been widely recognized as an effective quality metric in pathology laboratories. Atanda et al. (2017) conducted a teaching hospital audit in Nigeria and reported a mean analytic TAT of 3.6 ± 2 days, with 86.7% of reports completed within five working days, demonstrating that high performance is achievable even in resource-constrained environments with systematic workflow monitoring [4]. Similarly, Muvugabigwi et al. (2017) reported initial median TATs of 30 days for samples sent abroad, which decreased dramatically to a median of five days following the implementation of local capacity-building initiatives, highlighting the impact of infrastructural development and workflow optimization on diagnostic timeliness [5].

Recent audits from lower-middle-income countries and South Asian contexts further illustrate variability in TAT. A Nigerian histopathology service reported mean TATs of 7.5 ± 9.7 days for surgical biopsies, with delays arising at different stages, including grossing, processing, reporting, and transcription [6]. In Sri Lanka, audits from university diagnostic laboratories reported mean TATs ranging from 3.1 to 4.8 days for routine and complex biopsy specimens, demonstrating that appropriate workflow organization and adequate staffing can achieve acceptable TAT even in high-volume centers [7]. Furthermore, contemporary audits from India and Sri Lanka emphasize that TAT is influenced by multiple factors, including specimen complexity, ancillary testing requirements, staffing patterns, and automation in the laboratory [8,9].

These studies collectively highlight three important points: (1) benchmarking TAT provides an evidence-based method to evaluate laboratory performance; (2) local capacity-building with automation of laboratories, workflow optimization, and adequate staffing can significantly reduce TAT; and (3) even in resource-limited settings in most Bangladeshi laboratories, achieving TAT comparable to international standards is feasible with structured quality improvement initiatives.

At the Department of Pathology, Bangladesh Medical University (BMU), routine pathology reporting constitutes a substantial component of diagnostic services. The present study aims to benchmark TAT across different specimen types; identify bottlenecks in pre-analytic, analytic, and post-analytic phases; and provide baseline performance metrics to guide continuous quality monitoring, workflow optimization, and improved patient care. By comparing our findings with regional and international data such as Atanda et al. (2017), Muvugabigwi et al. (2017), and recent South Asian audits [4,5], we aim to situate our performance in a global context, highlighting strengths, gaps, and opportunities for improvement.

Audit standards

TAT is a key performance indicator for laboratories, representing the total time from sample reception to reporting results. A compliance standard is the rule or benchmark that a process must meet for a test to be considered acceptable. The target TAT benchmarks applied in this study were predefined for each test category, with a uniform compliance standard of at least 90% [10]. For histopathology, large, medium, and small specimens were expected to be reported within ≤8 working days. Cytopathology services, including fluid cytology, liquid-based cytology (LBC), and fine-needle aspiration cytology (FNAC), both superficial/non-guided and image-guided, had a target turnaround time of ≤5 working days. For specialized investigations, the target turnaround times were set at ≤20 working days for immunohistochemistry (IHC), ≤10 working days for direct immunofluorescence (DIF), and ≤12 working days for fluorescent in situ hybridization (FISH) [10].

## Materials and methods

Study design and setting

This retrospective study was conducted to evaluate TAT performance across different diagnostic modalities within the Department of Pathology. The audit covered a one-month period from April 1, 2025, to April 30, 2025, allowing assessment of routine departmental workflow under standard operational conditions. The study focused exclusively on in-house diagnostic services, reflecting real-world laboratory performance without external processing delays.

Data were retrieved from multiple validated sources, including the Laboratory Information System (LIS), manual laboratory registers, and departmental pathology reporting software, to ensure completeness and cross-verification of records. During the study period, a total of 2,899 completed cases that were received, processed, and reported within the department were included for final analysis.

Inclusion and exclusion criteria

All in-house pathology cases that were received, processed, and reported within the specified audit period were eligible for inclusion. This included cases with complete documentation of receipt time, processing milestones, and final report authorization.

Cases were excluded if they met any of the following criteria: (1) consultation or referral cases received from external institutions, due to variability in pre-analytical timelines; (2) frozen section cases, as these follow a different workflow and TAT benchmark; and (3) cases with missing, incomplete, or inconsistent time stamps in the LIS or manual records. These exclusion criteria were applied to maintain data accuracy, internal consistency, and comparability across diagnostic modalities.

Data collection method

Data were collected using a structured Microsoft Excel (Microsoft Corp., Redmond, WA, USA) spreadsheet specifically designed for the audit. The data collection tool incorporated predefined dropdown menus to standardize variable entries such as specimen type and diagnostic modality. Automated formulas were embedded to calculate TAT based on recorded timestamps, minimizing manual calculation errors.

Additionally, conditional formatting rules were applied to visually flag cases exceeding predefined TAT benchmarks, enabling rapid identification of out-of-TAT cases. Cross-checking between electronic and manual data sources was performed to ensure data integrity and completeness before final analysis.

TAT was defined as the interval between specimen receipt in the pathology laboratory and final report authorization in the reporting system. TAT was measured in hours or days, depending on the diagnostic modality, in accordance with departmental standard operating procedures.

Statistical analysis

Data were analyzed using descriptive statistical methods. Continuous variables, including TAT, were summarized using mean, median, standard deviation, and interquartile range (IQR) as appropriate based on data distribution. Categorical variables were expressed as frequencies and percentages.

Ethical considerations

Ethical approval was not required for this study, as it involved no direct patient contact and no additional procedures on patient samples beyond routine diagnostic testing. The study was conducted solely using retrospective data extracted from the LIS, including samples that had already been processed, reported, and signed out. All data were analyzed in an anonymized manner, ensuring patient confidentiality throughout the study.

## Results

Table 1 summarizes the TAT performance for histopathology specimens of different sizes, i.e., large, medium, and small. It includes the total number of cases reported; average, maximum, and minimum TAT in days; the number of reports completed within the target delivery time; and the corresponding percentage of reports meeting the TAT target. Overall, smaller specimens showed better TAT compliance, with 845 (89.51%) small specimen reports completed within the target timeframe compared to 412 (92.58%) for medium and 214 (77.10%) for large specimens.

**Table 1:** TAT performance for histopathology specimens. TAT: turnaround time

Parameter	Large specimen	Medium specimen	Small specimen
Total cases reported	214	445	944
Average TAT (days)	8	7	7
Maximum TAT (days)	28	23	21
Minimum TAT (days)	3	3	3
Reports within target delivery days	165	412	845
Percentage within TAT (%)	165 (77.10%)	412 (92.58%)	845 (89.51%)

Table 2 presents the TAT performance for various cytopathology specimen types, i.e., fluid, LBC, FNAC, and guided FNAC. It includes total cases reported; average, maximum, and minimum TAT in days; the number of reports completed within target delivery days; and the percentage of reports meeting the TAT target. Fluid specimens had the highest TAT compliance, 178 (96.22%), while fuided FNAC showed the lowest, 64 (86.48%). For LBC, it was 134 (93.06%), and for non-guided FNAC, it was 492 (88.81%).

**Table 2:** TAT performance for cytopathology specimens. TAT: turnaround time; LBC: liquid-based cytology; FNAC: fine-needle aspiration cytology

Parameter	Fluid	LBC	Non-guided FNAC	Guided FNAC
Total cases reported	185	144	554	74
Average TAT (days)	4	3	4	5
Maximum TAT (days)	15	10	59	9
Minimum TAT (days)	2	1	1	2
Reports within target delivery days	178	134	492	64
Percentage within TAT (%)	178 (96.22%)	134 (93.06%)	492 (88.81%)	64 (86.48%)

Table 3 shows the TAT performance for advanced laboratory tests, including IHC, DIF, and FISH. It presents total cases reported; average, maximum, and minimum TAT in days; the number of reports completed within target delivery days; and the percentage of reports meeting the TAT target. FISH achieved compliance at 9 (100%) within the target delivery days, while IHC achieved 157 (88.20%) and DIF achieved 103 (66.76%) compliance.

**Table 3:** TAT performance for specialized laboratory tests. TAT: turnaround time; IHC: immunohistochemistry; DIF: direct immunofluorescence; FISH: fluorescent in situ hybridization

Parameter	IHC	DIF	FISH
Total cases reported	178	152	9
Average TAT (days)	12	11	12
Maximum TAT (days)	38	19	14
Minimum TAT (days)	4	7	10
Reports within target delivery days	157	103	9
Percentage within TAT (%)	157 (88.20%)	103 (66.76%)	9 (100.0%)

Table 4 presents the TAT performance of various diagnostic tests performed in the laboratory, including histopathology, cytopathology, IHC, immunofluorescence, and cytogenetic diagnostics. For each test or sample type, the average TAT (measured in working days) and the percentage of reports delivered within the designated TAT are provided.

**Table 4:** Diagnostic test TAT and compliance summary. TAT: turnaround time; LBC: liquid-based cytology; IHC: immunohistochemistry; DIF: direct immunofluorescence; FISH: fluorescent in situ hybridization; FNAC: fine-needle aspiration cytology

Test category	Test name	Average TAT	% reports delivered within TAT
Histopathology	Large sample	8 working days	165 (77.10%)
Histopathology	Medium sample	7 working days	412 (92.58%)
Histopathology	Small sample	7 working days	845 (89.51%)
Cytopathology	Fluid Sample	4 working days	178 (96.22%)
Cytopathology	LBC	3 working days	134 (93.06%)
Cytopathology	Superficial FNAC	4 working days	492 (88.81%)
Cytopathology	Guided FNAC	5 working days	64 (86.48%)
Immunohistochemistry	IHC	12 days	157 (88.20%)
Immunofluorescence	DIF	11 working days	103 (67.76%)
Cytogenetic test	FISH	12 working days	9 (100.00%)

Table 5 summarizes the TAT performance for different sizes of histopathology specimens. Large specimens had the longest average and median TAT (~8 working days) and the widest IQR, reflecting greater processing complexity and additional ancillary testing. Medium and small specimens had slightly shorter TATs (~7 working days), with narrower IQRs indicating more consistent processing times. The proportion of reports completed within the target TAT increased as specimen size decreased, ranging from 77.10% for large specimens to 89.51% for small specimens.

**Table 5:** TAT performance for histopathology specimens. TAT is expressed in working days (WDs). TAT: turnaround time; IQR: interquartile range

Specimen type	Total cases	Average TAT (WDs)	Median TAT (WDs)	IQR (WDs)	Maximum TAT (WDs)	Reports within TAT (%)
Large specimen	214	8	~8	Wider IQR	28	77.10%
Medium specimen	445	7	~7	Moderate IQR	23	92.58%
Small specimen	944	7	~7	Narrow IQR	21	89.51%

Table 6 presents the TAT performance for various cytopathology tests. LBC had the shortest average TAT (three working days) with a narrow IQR and 93.06% of reports completed within the expected timeframe. Fluid cytology and superficial FNAC tests had slightly longer TATs (four working days) with narrow to moderate IQRs. Guided FNAC procedures had the longest average TAT among cytology tests (five working days) due to occasional repeat procedures or delayed clinical correlation. Overall, the majority of cytopathology reports were completed within the TAT, ranging from 86.48% to 96.22%.

**Table 6:** TAT performance for cytopathology specimens. TAT expressed in working days (WDs). TAT: turnaround time; LBC: liquid-based cytology; FNAC: fine-needle aspiration cytology; IQR: interquartile range

Test type	Total cases	Average TAT (WDs)	Median TAT (WDs)	IQR (WDs)	Maximum TAT (WD)	Reports within TAT (%)
Fluid cytology	185	4	≤4	Narrow	15	96.22%
LBC	144	3	≤3	Narrow	10	93.06%
FNAC – superficial	554	4	≤4	Moderate	59	88.81%
FNAC – guided	74	5	≤5	Moderate	9	86.48%

Table 7 shows TAT performance for specialized laboratory tests, including IHC, immunopathology, and molecular pathology tests. IHC and DIF tests had moderate to wide variability in TAT, with average/median values of 11-12 working days. Cytogenetic tests, such as FISH, had short and consistent TATs, with all reports completed within the target timeframe. The percentage of reports completed within TAT varied from 67.76% for DIF to 100% for molecular pathology tests.

**Table 7:** TAT performance for specialized laboratory tests. TAT expressed in working days (WDs). TAT: turnaround time; IQR: interquartile range; IHC: immunohistochemistry; DIF: direct immunofluorescence; FISH: fluorescent in situ hybridization

Test category	Test name	Total cases	Average TAT (WDs)	Median TAT (WDs)	IQR (WDs)	Maximum TAT (WDs)	Reports within TAT (%)
Immunohistochemistry	IHC	178	12	~12	Moderate	38	88.20%
Immunofluorescence	DIF	152	11	~11	Wide	19	67.76%
Cytogenetic	FISH	9	12	~12	Narrow	14	100.00%

Table 8 presents a side-by-side comparison of the standard TAT requirements and the observed laboratory performance across multiple diagnostic tests, including histopathology, cytopathology, FNAC, IHC, immunopathology, and molecular pathology. For each test type, the standard TAT, observed average TAT, compliance benchmark (≥90%), observed compliance, and compliance gap are provided to assess adherence to quality indicators.

**Table 8:** Comparison of standard versus observed TAT and compliance gap. TAT: turnaround time; WD: working day; LBC: liquid-based cytology; IHC: immunohistochemistry; DIF: direct immunofluorescence; FISH: fluorescent in situ hybridization; FNAC: fine-needle aspiration cytology

Test category	Test/Code	Standard TAT (WDs)	Observed average TAT (WDs)	Compliance standard	Observed compliance	Compliance gap (observed − standard)
Histopathology	Large sample	≤8	8	≥90%	77.10%	–12.90%
Histopathology	Medium sample	≤8	7	≥90%	92.58%	–2.58%
Histopathology	Small sample	≤8	7	≥90%	89.51%	–0.49%
Cytopathology	Fluid sample	≤5	4	≥90%	96.22%	+6.22%
Cytopathology	LBC	≤5	3	≥90%	93.06%	+3.06%
FNAC (cytopathology)	Superficial FNAC	≤5	4	≥90%	88.81%	–1.19%
FNAC (cytopathology)	Guided FNAC	≤5	5	≥90%	86.48%	–3.52%
Immunohistochemistry	IHC	≤20	12 days	≥90%	88.20%	–1.80%
Immunofluorescence	DIF	≤10	11	≥90%	67.76%	–22.24%
Cytogenetic study	FISH	≤12	12	≥90%	100%	+10.00%

In histopathology, all three specimen groups (large, medium, and small) met the standard TAT requirement; however, compliance remained below the expected threshold, particularly for large specimens (-12.90%). Cytopathology performed strongly, with both fluid samples and LBC exceeding the compliance standard (+6.22% and +3.06%, respectively). Superficial and guided FNAC met TAT targets but showed slight compliance deficits. Specialized tests demonstrated variable performance. IHC achieved a favorable average TAT well within the 20-day limit, yet its compliance fell marginally short of the 90% standard (-1.80%). DIF showed the largest deviation, with both TAT and compliance substantially below expectations (-22.24% compliance gap), representing the most significant performance concern. Conversely, cytogenetic tests (FISH) showed excellent performance, achieving 100% compliance and remaining well within their respective TAT standards.

## Discussion

TAT is a widely accepted surrogate marker of laboratory efficiency and diagnostic quality in anatomic pathology, with direct implications for clinical decision-making, patient safety, and healthcare system performance [11,12]. This study provides a detailed benchmarking analysis of TAT across routine histopathology, cytopathology, and specialized diagnostic services at the Department of Pathology, BMU, offering institution-specific insights within an internationally relevant quality framework.

Although all histopathology specimen categories in this study met the institutional standard of ≤8 working days in terms of mean TAT, compliance with the ≥90% benchmark varied significantly by specimen size. Large specimens demonstrated the longest median TAT and the widest IQR, indicating substantial variability in processing time. This may partly reflect the complexity of large surgical specimens such as breast or gastrointestinal resections, which typically require extensive grossing, higher block numbers, and additional ancillary procedures. Similar observations have been reported in prior studies, where specimen complexity, increased block numbers, prolonged fixation times, and ancillary testing were shown to be key contributors to delayed reporting in large surgical specimens [13-15].

The progressively improved compliance observed in medium and small specimens aligns with international findings that reduced tissue volume and standardized grossing protocols lead to more predictable workflows and improved TAT performance [16]. These results emphasize that average TAT alone may mask workflow inefficiencies, and that variability measures such as IQR and compliance rates are critical for accurate performance assessment, particularly in high-volume academic laboratories [17].

Cytopathology services demonstrated superior TAT performance, with average reporting times between three and five working days and compliance rates exceeding 86% across all test types. Fluid cytology and LBC surpassed the ≥90% benchmark, reflecting streamlined specimen processing and rapid interpretive workflows. Previous multicenter studies have similarly documented cytopathology as one of the fastest diagnostic services due to minimal laboratory processing requirements and reduced reliance on ancillary testing [18].

The modest compliance shortfall observed in non-guided and guided FNAC procedures appears to be influenced primarily by pre-analytical and clinical factors, including repeat aspirations, inadequate patient’s medical information, missing radiologic data, and delayed clinicopathologic correlation. The markedly prolonged maximum TAT observed in a few non-guided FNAC cases likely reflects operational or patient-related delays rather than routine laboratory performance. The majority of cases clustered within the IQR. This pattern has been well documented in the literature, where FNAC TAT variability is often driven by factors outside the laboratory’s direct control rather than internal inefficiency [19].

Specialized laboratory tests showed the greatest heterogeneity in both TAT and compliance, reflecting differences in assay complexity and workflow dependency. Cytogenetic tests (FISH) achieved 100% compliance and remained well within standard TAT limits. This finding is consistent with reports from high-throughput molecular laboratories where standardized protocols, batch-independent workflows, and defined reporting pathways enable predictable TATs despite technical sophistication [20,21].

In contrast, DIF emerged as the poorest-performing service, with a compliance rate of only 67.76% and the largest negative compliance gap (-22.24%). International studies have identified similar challenges in DIF services, attributing delays to reagent sensitivity, special stains in addition to hematoxylin and eosin stain for renal samples, limited technical expertise, batch processing practices, and dependency on fresh or appropriately preserved specimens [5]. The wide IQR observed in this study further supports the presence of structural workflow bottlenecks rather than random variation.

IHC demonstrated acceptable average TAT well within the 20-day standard but narrowly missed the ≥90% compliance benchmark. This mirrors findings from CAP Q-Probes and other quality improvement studies, which highlight that while IHC TAT averages are generally satisfactory, variability often arises from antibody availability, repetitions of tests due to technical errors, reflex testing algorithms, and workload surges in oncologic cases [22,23].

The comparative analysis of standard versus observed TAT highlights a critical distinction between achieving acceptable mean TATs and maintaining consistent compliance. Several services met standard TAT thresholds yet failed to achieve ≥90% compliance, underscoring the importance of variability reduction rather than reliance on average metrics alone. International accreditation frameworks, including ISO 15189 and the CAP Laboratory Accreditation Program, increasingly emphasize percentile-based and compliance-driven indicators for this reason [24,25].

Targeted interventions such as specimen complexity-based workflow stratification, dedicated processing lines for large histopathology specimens, introducing automation in laboratories, scheduling for DIF services, and real-time TAT monitoring dashboards may substantially improve compliance. Furthermore, the use of median and IQR-based reporting, as adopted in this study, aligns with best-practice recommendations for analyzing right-skewed laboratory performance data [26].

This study represents one of the most comprehensive TAT benchmarking analyses across routine and advanced pathology services in a tertiary academic center in Bangladesh. By integrating compliance gaps with international standards, it provides actionable data for continuous quality improvement and contributes valuable regional evidence to the global pathology quality literature.

However, the audit had several limitations. The study period was short. Delays in large histopathology samples were mainly due to fixation issues, the need for re-grossing, and insufficient clinical information. At the same time, repeated aspirations stemming from limited resident experience affected the timeliness of superficial FNAC reporting. Inadequate clinical details also influenced delays in guided FNAC. IHC and DIF services faced setbacks from equipment downtime, reagent shortages, technical errors requiring re-staining, and the absence of a prioritization protocol for urgent cases. Additionally, intra-departmental consultations and integration of residency training within the laboratory workflow further extended analytic TAT. To overcome these limitations, dedicated staffing for DIF and FISH tests, automated LIS alerts for overdue reports, early IHC ordering, and strict prioritization protocols are recommended. Strengthening quality control practices, ensuring staff competency in special stains, and maintaining adequate inventory, including safety stock of critical reagents, are essential to minimize technical delays. Furthermore, implementing systematic root-cause analysis for any TAT deviation and fostering close communication among pathologists, laboratory technologists, and clinicians will help identify bottlenecks promptly and ensure smoother workflow across departments.

## Conclusions

From specimen receipt to diagnostic signature, pathology services at BMU demonstrate strong overall performance, particularly in cytopathology and cytogenetic diagnostics. However, variability in histopathology, especially for large specimens, and significant delays in DIF represent key opportunities for targeted process optimization. Addressing these gaps through structured quality improvement initiatives will enhance diagnostic efficiency, consistency, and, ultimately, patient care outcomes.
